# Evidence of recombination in Hepatitis C Virus populations infecting a hemophiliac patient

**DOI:** 10.1186/1743-422X-6-203

**Published:** 2009-11-18

**Authors:** Pilar Moreno, Macarena Alvarez, Lilia López, Gonzalo Moratorio, Didier Casane, Matías Castells, Silvia Castro, Juan Cristina, Rodney Colina

**Affiliations:** 1 Laboratorio de Virología Molecular, Centro de Investigaciones Nucleares, Facultad de Ciencias, Universidad de la República, Montevideo, Uruguay; 2 Servicio Nacional de Sangre, Montevideo, Uruguay; 3 Laboratoire Evolution Génomes Spéciation, CNRS 91198 Gif-sur-Yvette, France; 4 Cátedra de hemoterapia, Hospital de Clínicas, Montevideo, Uruguay

## Abstract

**Background/Aim:**

Hepatitis C virus (HCV) infection is an important cause of morbidity and mortality in patients affected by hereditary bleeding disorders. HCV, as others RNA virus, exploit all possible mechanisms of genetic variation to ensure their survival, such as recombination and mutation. In order to gain insight into the genetic variability of HCV virus strains circulating in hemophiliac patients, we have performed a phylogenetic analysis of HCV strains isolated from 10 patients with this kind of pathology.

**Methods:**

Putative recombinant sequence was identified with the use of GARD program. Statistical support for the presence of a recombination event was done by the use of LARD program.

**Results:**

A new intragenotypic recombinant strain (1b/1a) was detected in 1 out of the 10 hemophiliac patient studied. The recombination event was located at position 387 of the HCV genome (relative to strain AF009606, sub-type 1a) corresponding to the *core *gene region.

**Conclusion:**

Although recombination may not appear to be common among natural populations of HCV it should be considered as a possible mechanism for generating genetic diversity in hemophiliacs patients.

## Background

Hepatitis C virus (HCV) is estimated to infect 170 million people worldwide and is the major causative agent of post transfusional hepatitis and parenterally transmitted sporadic non A non B Hepatitis [1]. Hemophiliacs and other patients with inherited bleeding disorders treated with non-inactivated clotting factor concentrates prior to the mid 1980 are at particular risk for acquiring HCV infection. Each clotting factor concentrated were made from plasma pools prepared from approximately 20000 blood donors at a time and this provided a single unit risk of infection of 5%. The introduction of inactivation procedures and blood donor screening for HCV has dramatically improved the safety of pooled plasma products [2,3]. In Uruguay hemophiliac patients were treated with fresh frozen plasma in the 60 s and with cryoprecipitate (VIII factor and fibrinogen rich fraction plasma) since the 70 s, these transfusions were responsible for most of hepatitis C transmission in hemophiliac. Since the 70 s and mid 80 s, industrial non-inactivated clotting factor was available in the world but this product was not used in Uruguay until the 90 s [4].

HCV is a member of the family *Flaviviridae*. HCV is a single stranded, positive sense, RNA virus with a genome of approximately 9400 bp [1,5]. Comparison of nucleotide sequences of variants recovered from different individuals around the world has revealed the existence of at least six major genetic groups and an increasing number of subtypes [1,6-8]. Since hemophiliacs were infected by clotting factors concentrates manufactured from many thousands of blood donors, the HCV genotype distribution may reflect that of the donor population. Besides, different HCV genotypes may have infected the same patient [3,9,10].

HCV as others RNA virus exploit all possible mechanisms of genetic variation to ensure their survival. The high rate of mutation generated by the polymerase and the high rate of replication of this virus results in the circulation *in vivo *of complex population of different but closely related viral variants, commonly referred to as a quasispecies [11-13]. It is known that recombination plays a significant role in the evolution of RNA viruses by creating genetic variation. Both inter and intragenotypic recombination have been reported in HCV populations in different geographic locations like Russia (2 k/1b) [14,15], Peru (1a/1b) [16], Vietnam (2/6) [17], Philippines (2b/1b) [18], France (2/5) [19], Uzbekistan (2 k/1b) [20], Japan (1a/1c) [21] and Ireland [22]. Intra-patient recombination has also been reported [23,24].

In order to gain insight into the possible role of recombination in shaping the HCV evolution in hemophiliac patients, we have performed a phylogenetic analysis of HCV strains circulating in Uruguayan patients with clinical diagnosis of this disease. The results of these studies revealed for the first time the presence of a natural intragenotypic HCV recombinant strain circulating in a Uruguayan hemophiliac patient.

## Results

**Figure 1:**
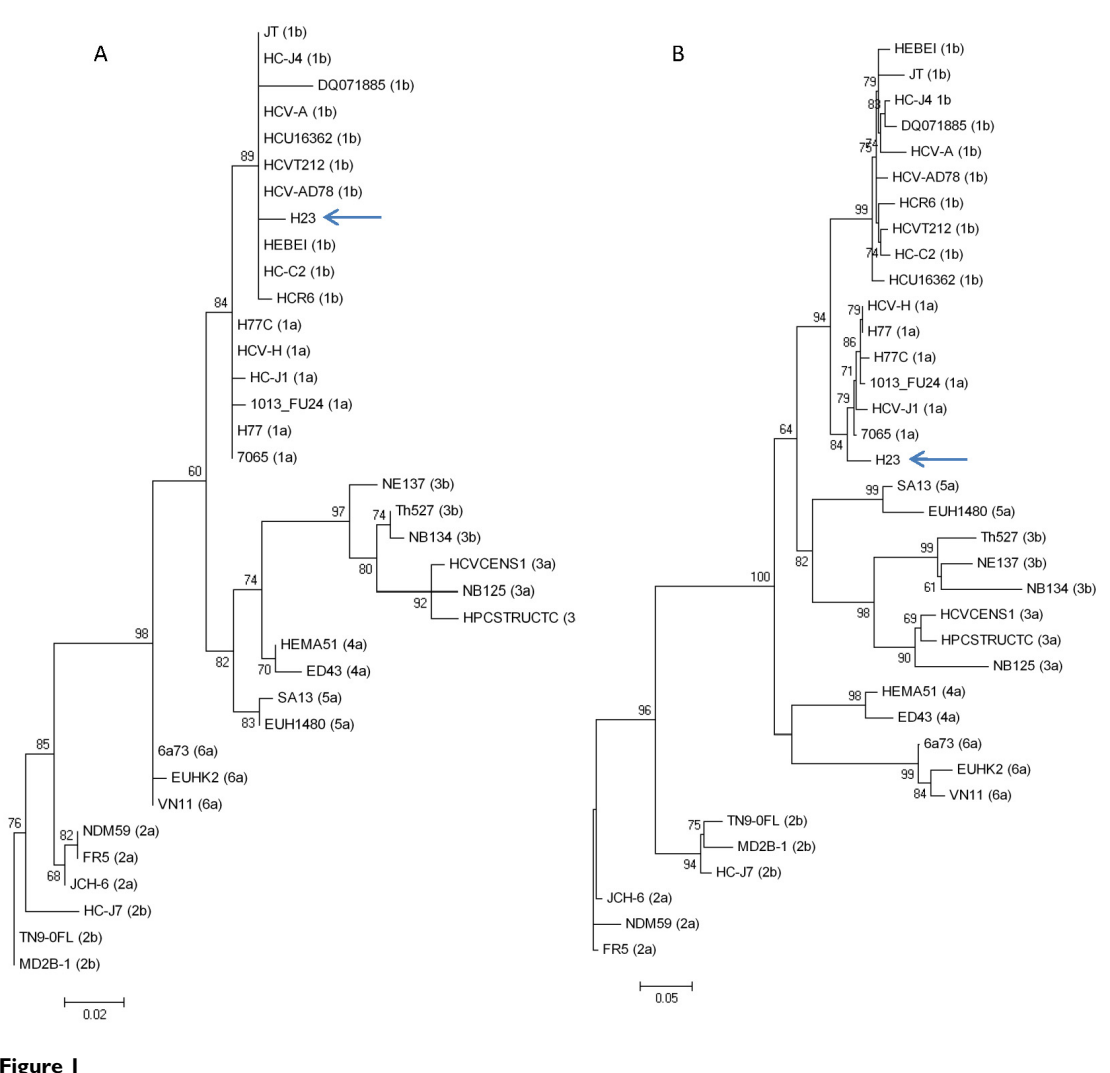
**Maximum likelihood phylogenetic tree analysis of HCV strains using the GTR plus gamma model**. Strains in the tree are shown by their accession number and their genotypes are indicated between parentheses for strains previously described (for accession numbers, genotypes and geographic origin of isolation see Table 1). Strain H23, isolated from a Uruguayan hemophiliac patient is shown by name. Numbers at each branch of the tree show aLRT values. Bars at the bottom of the trees show distance. The phylogeny for the *5'NCR *and *core *region is shown in (A) and (B) respectively.

To gain insight into the genetic variability of HCV strains circulating in Uruguayan hemophiliac patients, we first obtained nucleotide sequences from five different genomic regions of the HCV genome from strains circulating in these patients. These sequences were aligned with corresponding sequences from 35 HCV strains of all types and sub-types isolated in different geographic regions of the world, obtained using the HCV LANL database [25]. The origin of the sequences and the strains used are listed in Table 1. Sequences were aligned using the CLUSTAL W program [26]. Once aligned, we first determined the evolutionary model that best fit our sequence data. Akaike Information Criteria and Hierarchical Likelihood Ratio Test showed that the General Time Reversible (GTR) plus gamma model best fit our sequence data. Using this model, maximum likelihood phylogenetic trees were created for the *5'NCR *and the *core *region. The results of these studies are shown in Figure 1. All strains in the tree are assigned according with their genotype. Each cluster is supported by very high aLRT values (see Figure 1). Interestingly, Uruguayan strain H23, assigned to subtype 1b in the *5'NCR *region, is assigned to subtype 1a in the *core *region (compare Figs. 1A and 1B) [GenBank: EU934902-EU934907].

**Table 1:** Origins of hepatitis C virus strains from LANL database.

Strain Name	Genotype	Geographic location	Accession number
HCV-A	1b	Australia	AJ000009

JT	1b	Japan	D11355

HCV-AD78	1b	Germany	AJ132996

HC-J4	1b	Japan	D00832

HCR6	1b	Japan	AY045702

HC-C2	1b	China	D10934

DQ071885	1b	Taiwan	DQ071885

HCU16362	1b	Korea	U16362

HCVT212	1b	Japan	AB049099

HEBEI	1b	China	L02836

H77C	1a	United States	AF011751

HCV-H	1a	United States	M67463

HC-J1	1a	Japan	D10749

1013_FU24	1a	United States	EU362876

7065	1a	United States	EF407455

H77	1a	United State	AF009606

FR5	2a	France	L38334

NDM59	2a	Japan	AF169005

JCH-6	2a	Japan	AB047645

HC-J7	2b	Japan	D10077

MD2B-1	2b	Japan	AF238486

TN9-0FL	2b	United State	DQ430815

HPCSTRUCTC	3a	France	L12355

NB125	3a	India	AY231596

HCVCENS1	3a	Germany	X76918

NB134	3b	India	AY231588

Th527	3b	Thailand	D37839

NE137	3b	Nepal	D16616

ED43	4a	Egypt	Y11604

HEMA51	4a	Japan	D45193

SA13	5a	South Africa	AF064490

EUH1480	5a	United Kingdom	Y13184

VN11	6a	Vietnam	L38339

EUHK2	6a	China	Y12083

6a73	6a	China	DQ480517

The same studies were done for the *E2*, *NS5A *and *NS5B *regions of the HCV genome. The results of these studies revealed that Uruguayan strain H23 is assigned to subtype 1a in these regions (data not shown).

**Figure 2:**
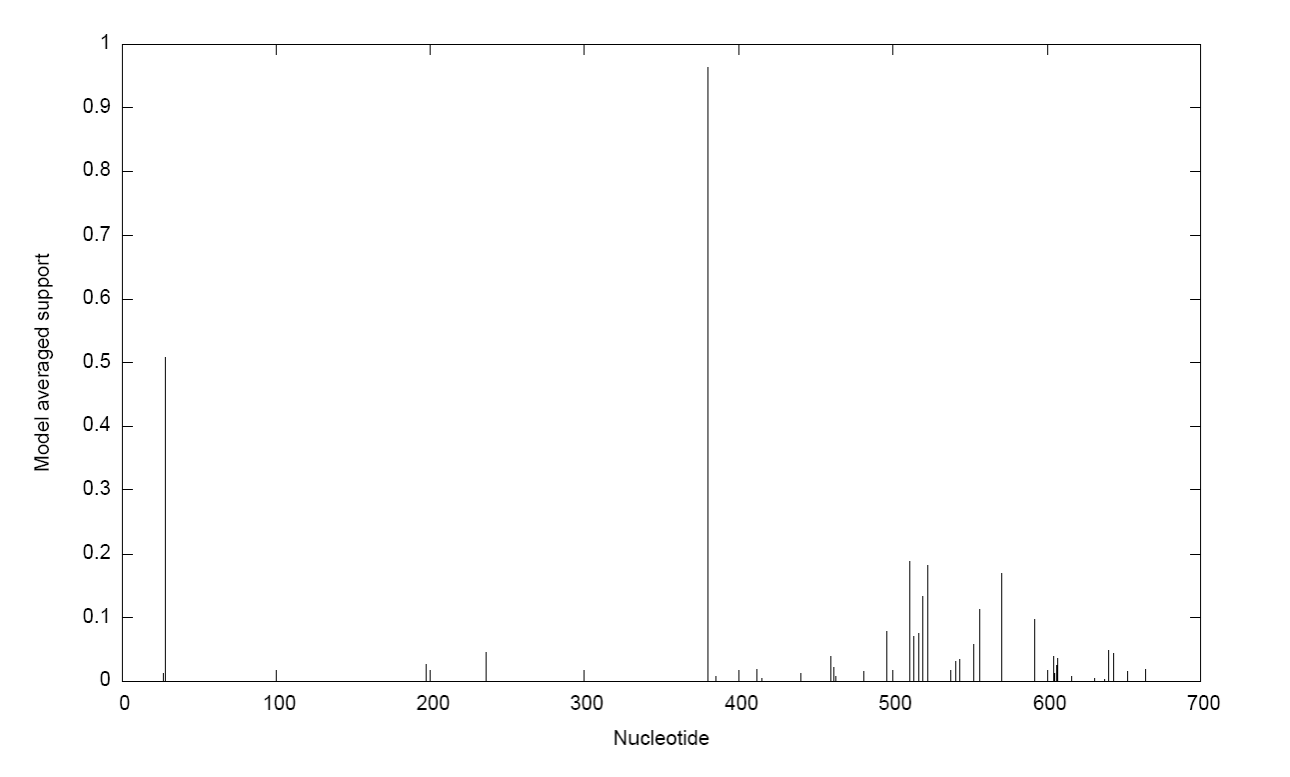
**Recombination break-point detection using GARD**. Support probability for inferred recombination break-points is shown on the left side of the figure. The nucleotide position in the alignment is shown on the *x*-axis of the graph.

To rule out that this results were the consequence of the co-infection of the patient with two different HCV subtypes and not really a recombinant strain, we amplified by PCR the *5'NCR-core *region of HCV strain H23 (from nucleotide 9 to 706, relative to the genome of HCV strain AF009606). Once amplified, sequences from that region of the genome of strain H23 were obtained by direct sequencing of the PCR fragment using the same primers used for amplification. Sequences from the *5'NCR-co*re region of HCV H23 strain were aligned with corresponding sequences from the genotype 1 HCV strains shown in Figure 1 using CLUSTAL W program [26] (see also Table 1). Once aligned, we used GARD to determine the presence of possible recombination break-points [27]. A recombination break-point is observed at position 379 of the alignment, representing position 387 of the HCV genome which corresponds to position 46 of HCV *core *gene (relative to strain AF009606) (see Figure 2). Two putative parental-like strains D11355 (subtype 1b) and AF009606 (subtype 1a) were identified.

**Figure 3:**
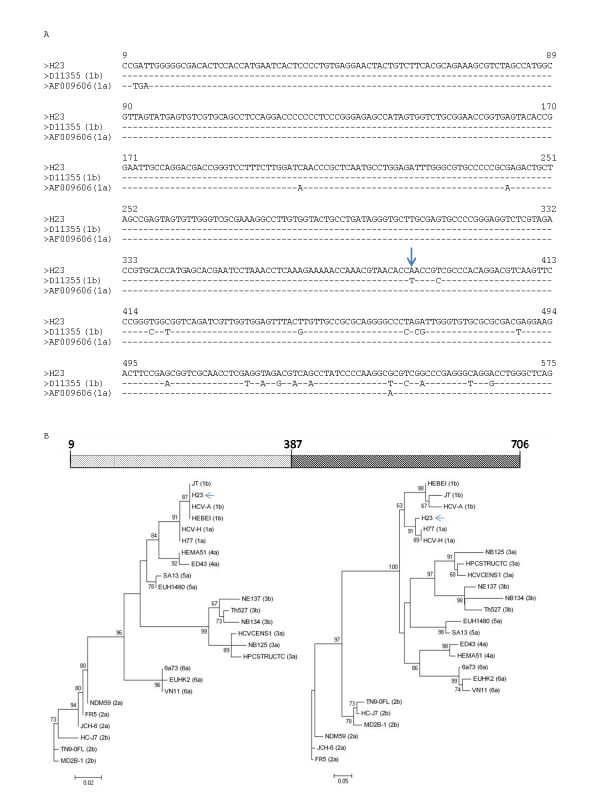
**Identification of recombination break-point in Uruguayan H23 HCV strain**. In (A) an alignment of 5'NCR plus *core *sequences of strains H23 and parental-like strains D11355 (sub-type 1b) and AF009606 (sub-type 1a) is shown. Identity to H23 is shown by a dash. Recombination break-point identified by GARD is shown by an arrow. In (B) a scheme representing H23 *5'NCR *plus *core *region sequences showing the recombination break-point is shown in the upper part of the figure. Numbers indicate nucleotide positions relative to strain AF009606. Maximum likelihood phylogenetic trees obtained using partial alignment before and after the recombination break-point are shown behind the scheme. Strains previously described are shown by their accession number and their genotype is indicated between parenthesis (see also Table 1). Strain H23 is shown by name and indicated by an arrow. Numbers at each branch of the trees show aLRT values. Bars at the bottom of the trees show distance.

In order to confirm the results obtained using the GARD approach, sequence alignment between H23 and its putative parental-like strains D11355 (subtype 1b) and AF009606 (subtype 1a) around the breakpoint were performed and maximum likelihood phylogenetic trees were constructed for the sequence alignment before and after the identified breakpoint. The results of these studies are shown in Figure 3. As it can be seen in the figure 3B, Uruguayan H23 strain is assigned to subtype 1b using the alignment from positions 9 to 386, and to subtype 1a from positions 387 to 706. To confirm that the recombination model we obtained gave a significant better fit to the data than the null hypothesis of no recombination, we used LARD program [28] employing a different dataset of putative parental-like HCV 1a and 1b strains. The results of these studies are shown in Figure S1 (see Additional File 1). Simulations of sequence evolution under the null hypothesis (i.e., no recombination) gave strong statistical support for the alternative hypothesis of recombination (*P *< 0.001).

To further characterize the presence of the recombination event in Uruguayan HCV H23 strain by other methods, we employed the Genotyping Tool of NCBI server [29]. As it can be seen in Figure S2 (see Additional File 2) and in Table S3 (see Additional File 3), the BLAST scores obtained in this analysis show the presence of a recombination event in HCV H23 strain.

## Discussion

Previous results have consistently given congruent results for HCV genotyping using different genomic regions [8]. In this study, incongruent results were found for one HCV strain circulating in a hemophiliac patient when different genomic regions were used (see Figure 1). This apparent discrepancy was due to the fact of the presence of a recombination break-point in HCV Uruguayan strain H23 at position 387 of HCV genome (relative to strain AF009606) (see Figure 2). Maximum likelihood phylogenetic tree analysis were performed using partial alignments of the same sequences before and after the identified break-point which support the presence of a recombination event at this position (see Figure 3). Moreover, this recombination model has a better statistically support against the null hypothesis of no recombination (see Figure S1 in Additional File 1). Furthermore, using a different set of putative parental-like strains, the Genotyping Tool results revealed BLAST scores that also support the recombination event (see Figure S2 in Additional File 2 and Table S3 in Additional File 3).

Previous HCV phylogenetic studies have shown that recombination breakpoints can be detected in non structural and structural regions of the HCV genome [14-22]. Interestingly, the recombination breakpoint identified in Uruguayan strain H23 is located inside the *core *Region.

Although recombination does not seem to be a common event in the evolution of the HCV populations, the results of these studies reveal that recombination can be considered as an evolutionary mechanism for generating genetic diversity in HCV populations circulating in Hemophiliac patients. Due to the fact that these Hemophiliac patients have been transfused many times, the possibility of being infected by two different HCV genotypes and subtypes is higher than in other kind of patients, increasing the possibility of a recombination event to take place.

## Conclusion

Given the implications of recombination for virus evolution and the development of vaccines, virus control programs, patient management and antiviral therapies, it is clearly important to determine the extent to which recombination plays a role in HCV evolution. This study demonstrates the presence of a recombinant 1b/1a HCV strain circulating in a hemophilic patient. Further studies will be needed in order to establish the role of recombination events in shaping the HCV evolution in hemophiliac patients.

## Methods

### Serum samples

Serum samples were obtained from 10 Hemophiliac patients with serological markers for HCV from the Hospital de Clínicas (Montevideo, Uruguay). Patients were screened using Riba-ELISA according to the manufacturer's instructions.

### RNA extraction, cDNA synthesis and amplification

HCV RNA was extracted from 140 μl serum samples with the QIAamp viral RNA Kit (QIAgen) according to the manufacturer's instruction. The extracted RNA was eluted from the columns with 50 μl RNAse free water. cDNA synthesis and PCR amplification of the 5' non-coding region (*5'NCR*), *core, E2, NS5a *and *NS5b *were carried out as previously described [30,31]. The *5'NCR-core *PCR from strain H23 (from nucleotide 9 to 706, relative to the genome of HCV strain AF009606) was performed as previously described [32]. Amplicons were purified using the DNA extraction Kit (Fermentas).

### Sequencing

The same primers of the PCR were used for sequencing the PCR fragments of each region. The sequencing reaction was done in both strands, and was carried out using the Big Dye DNA sequencing Kit (Perkin Elmer) on a DNA sequencing apparatus ABI3130 (Perkin Elmer).

### Sequence analysis

*5'NCR*, *core, E2, NS5a *and *NS5b *sequences obtained from Uruguayan hemophiliac patients were aligned with corresponding sequences from strains from all HCV genotypes and sub-types, isolated in different geographic regions of the world. Sequences were obtained using the HCV LANL database (see Table 1) [25]. Sequences were aligned using the CLUSTAL W program [26]. Once aligned, the best evolutionary model that describe our sequence data was assessed using the Modelgenerator [33] (Akaike Information Criteria and Hierarchical Likelihood Ratio test indicated that the GTR plus gamma model was the most appropriate model to represent the sequence data). Using this model, maximum likelihood phylogenetic trees were constructed using the PhyML program [34,35]. As a measure of the robustness of each node, we employed an approximate Likelihood Ratio Test (aLRT), that assesses that the branch being studied provides a significant likelihood gain, in comparison with the null hypothesis that involves collapsing that branch but leaving the rest of the tree topology identical [36].

### Genetic Algorithm Recombination Detection (GARD) Analysis

To detect possible recombination events we used GARD method (available at http://www.datamonkey.org/GARD/) for detecting discordant phylogenetic signal in sequence alignments, which provides estimates of the number and location of break points and segment-specific phylogenetic trees [27].

### Genotyping tool at NCBI

To detect possible HCV recombinant strain sequences, we used Genotyping Tool at the National Center for Biotechnology Information (available at: http://www.ncbi.nih.gov/projects/genotyping/formpage.cgi). This approach uses BLAST to compare a query sequence to a set of HCV reference sequences of known genotypes and sub-types. The query sequence is broken into segments for comparison to the reference so that the mosaic organization of recombinant sequences could be revealed [29].

### LARD (Likelihood Analysis of Recombination in DNA) analysis

To assess whether the recombination model we obtained gave a significantly better fit to the data than the null hypothesis of no recombination, we used LARD program [28]. Briefly, for every possible breakpoint, the sequence alignment was divided into two independent regions for which the branch lengths of a tree of the putative recombinant and its two parent sequences were optimized. The two results (likelihoods) obtained by using the separate regions were then combined to give a likelihood score for that breakpoint position and the breakpoint position that yielded the highest likelihood then was compared, by using a likelihood ratio test, to the likelihood obtained from the same data under a model that permitted no recombination. The likelihood ratio obtained by using the real data were evaluated for significance against a null distribution of likelihood ratios produced by using Monte Carlo simulation of sequences generated without recombination. Sequences were simulated 1,000 times by using the maximum likelihood model parameters and sequence lengths from the real data using Seq-Gen [37].

## List of Abbreviations

aLTR: approximate Likelihood Ratio Test; GARD: Genetic Algorithm Recombination Detection Analysis; GTR: General Time Reversible; HCV: Hepatitis C Virus; IFN: Interferon; LARD: Likelihood Analysis of Recombination in DNA; *5'NCR*: 5'Non Coding Region.

## Competing interests

The authors declare that they have no competing interests.

## Authors' contributions

PM and MA conceived the study. PM, LL, SC and GM designed the analysis. JC and PM performed the recombinant studies. DC performed the LARD analysis. JC and RC contributed to the discussion of all results found in this work. PM and RC wrote the paper. All authors read and approved the final manuscript.

## Supplementary Material

Additional file 1 **Distribution of the likelihood ratios expected by chance**. An alignment of the *5'NCR *plus *Core *region of the sequences corresponding to strain H23 and putative parental like strains D11355 (sub-type 1b), and AF009606 (sub-type 1a) was used in this study. The distribution of the likelihood ratios for the null hypothesis (i. e. no recombination) is shown. The y-axis shows the number of simulations. Likelihood ratios are shown at the bottom of the figure. The arrow show the likelihood ratio obtained for the real dataset for the putative recombinant strain. Click here for file

Additional file 2 **Results of Genotyping tool at NCBI**. A graphic output of the analysis of 1b/1a recombinant strain H23 using a window of 200 bases and a movement of 10 bases is show. In the upper part of the figure a schematic representation of the mosaic H23 strain sequences is shown and the colors indicate the corresponding HCV subtype identified by NCBI database. BLAST scores are indicated in the left side of the figure. Positions in the sequence alignment are shown at the bottom. Colors correspond to the different HCV subtypes and are indicated at the right side of the figure. See also Table S3 (Additional File 3). Click here for file

Additional file 3 **Table with results for H23 strain using Genotyping Tool at NCBI**. The table shows the results for H23 strain using Genotyping Tool as implemented in NCBI. The position alignment, the blast score, the genotype and G.I. of the reference strain are indicated. Click here for file
