# Supplementary figures and images for: Evidence of recombination in Hepatitis C Virus populations infecting a hemophiliac patient
Source: Virol J. 2009 Nov 18;6:203. doi: 10.1186/1743-422X-6-203 (PMC2784780; doi:10.1186/1743-422X-6-203)

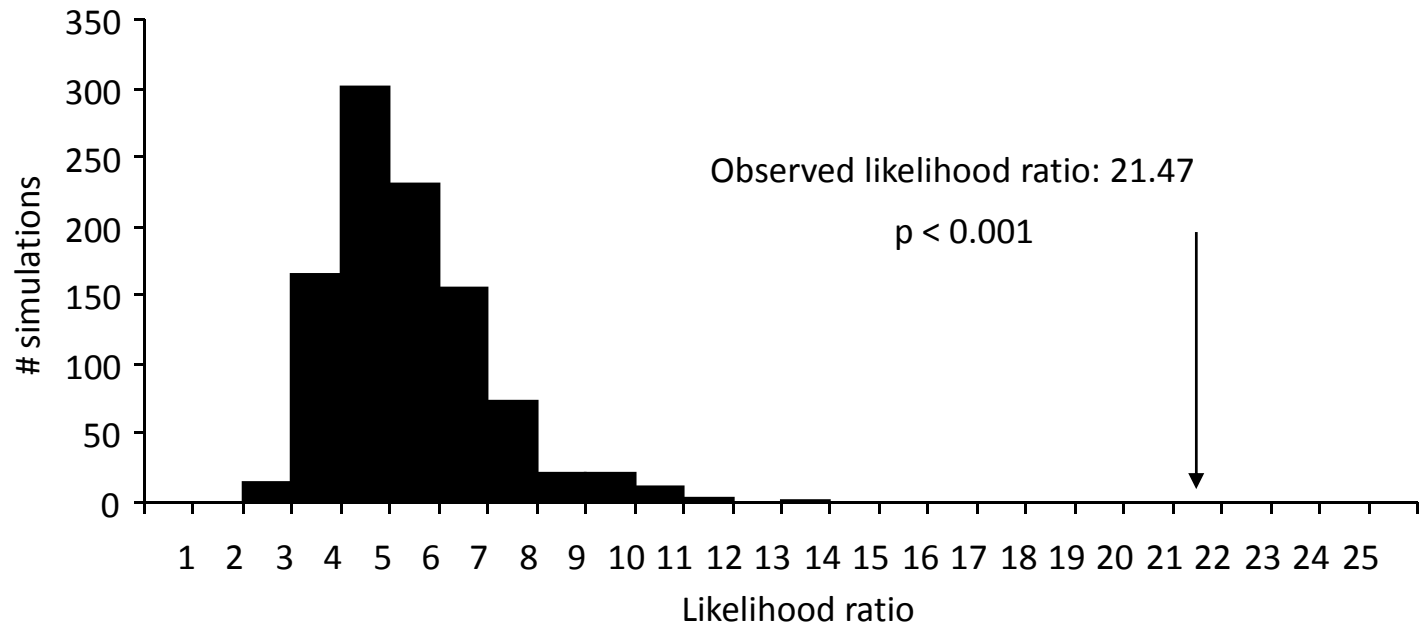

Supplement: Additional file 1 — Distribution of the likelihood ratios expected by chance. An alignment of the 5'NCR plus Core region of the sequences corresponding to strain H23 and putative parental like strains D11355 (sub-type 1b), and AF009606 (sub-type 1a) was used in this study. The distribution of the likelihood ratios for the null hypothesis (i. e. no recombination) is shown. The y-axis shows the number of simulations. Likelihood ratios are shown at the bottom of the figure. The arrow show the likelihood ratio obtained for the real dataset for the putative recombinant strain. [file 1743-422X-6-203-S1.PDF]

## window borders

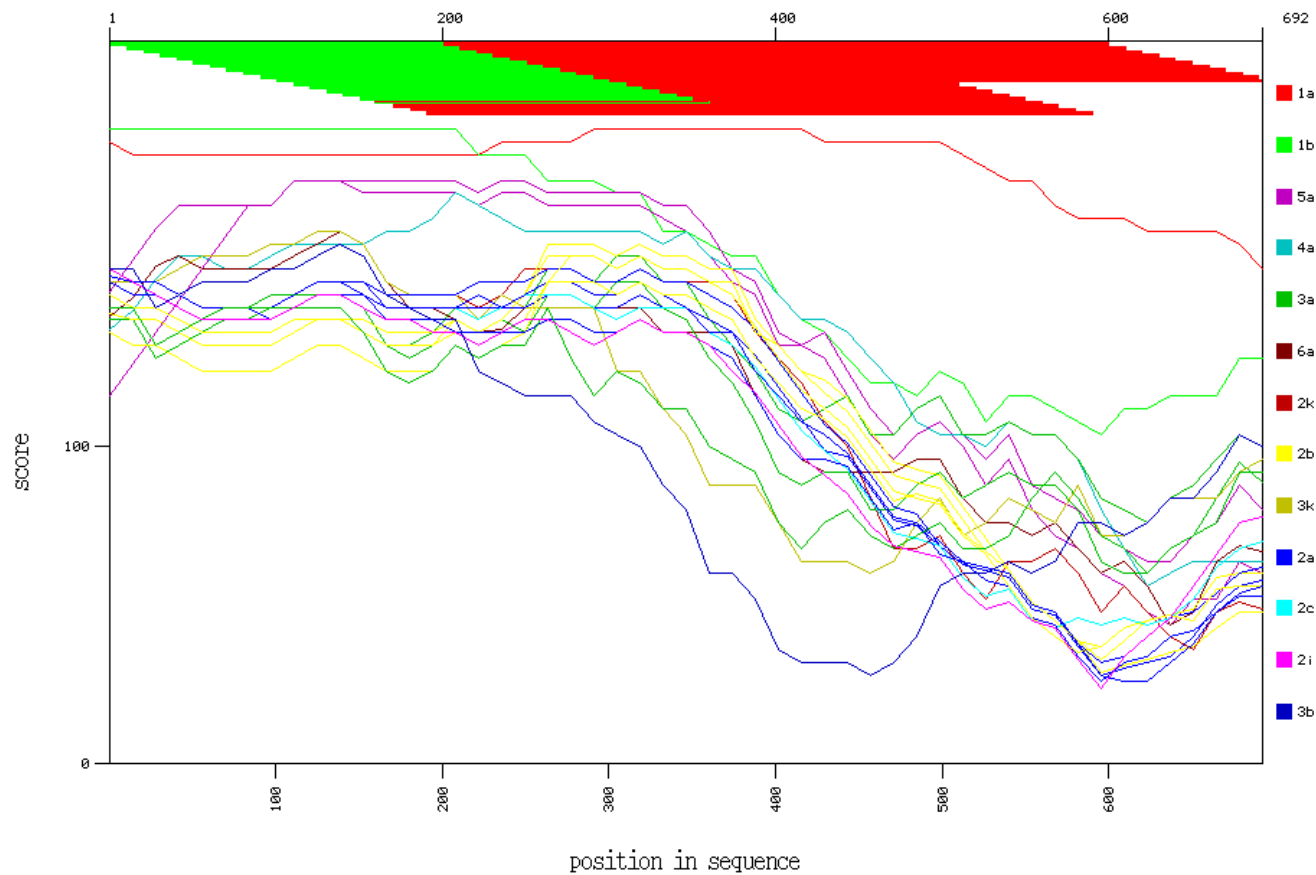

Supplement: Additional file 2 — Results of Genotyping tool at NCBI. A graphic output of the analysis of 1b/1a recombinant strain H23 using a window of 200 bases and a movement of 10 bases is show. In the upper part of the figure a schematic representation of the mosaic H23 strain sequences is shown and the colors indicate the corresponding HCV subtype identified by NCBI database. BLAST scores are indicated in the left side of the figure. Positions in the sequence alignment are shown at the bottom. Colors correspond to the different HCV subtypes and are indicated at the right side of the figure. See also Table S3 (Additional File 3). [file 1743-422X-6-203-S2.PDF]
