# Supplementary material for: Evidence of recombination in Hepatitis C Virus populations infecting a hemophiliac patient
Source: Virol J. 2009 Nov 18;6:203. doi: 10.1186/1743-422X-6-203 (PMC2784780; doi:10.1186/1743-422X-6-203)
Supplement: Additional file 3 — Table with results for H23 strain using Genotyping Tool at NCBI. The table shows the results for H23 strain using Genotyping Tool as implemented in NCBI. The position alignment, the blast score, the genotype and G.I. of the reference strain are indicated. [file 1743-422X-6-203-S3.DOC]

**_______________________________________________________________________________**

**Nucleotide Genotype G.I. of**

**Alignment in middle BLAST of reference reference**

**Window No. positions of window score strain strain**

**_______________________________________________________________________________**

1 1-200 100 191 1b 221614

2 10-210 110 196 1b “

3 20-220 120 196 1b “

4 30-230 130 200 1b “

5 40-240 140 200 1b “

6 50-250 150 200 1b “

7 60-260 160 200 1b “

8 70-270 170 200 1b “

9 80-280 180 200 1b “

10 90-290 190 200 1b “

11 100-300 200 200 1b “

12 110-310 210 200 1b “

13 120-320 220 200 1b “

14 130-330 230 200 1b “

15 140-340 240 200 1b “

16 150-350 250 200 1b “

17 160-360 260 200 1b “

18 170-370 270 200 1b “

19 180-380 280 196 1b “

**_______________________________________________________________________________**

**Nucleotide Genotype G.I. of**

**Alignment in middle BLAST of reference reference**

**Window No. positions of window score strain strain**

**_______________________________________________________________________________**

20 190-390 290 192 1a 2316097

21 200-400 300 196 1a “

22 210-410 310 196 1a “

23 220-420 320 196 1a “

24 230-430 330 196 1a “

25 240-440 340 200 1a “

26 250-450 350 200 1a “

27 260-460 360 200 1a “

28 270-470 370 200 1a “

29 280-480 380 200 1a “

30 290-490 390 200 1a “

31 300-500 400 200 1a “

32 310-510 410 200 1a “

33 320-510 420 200 1a “

34 330-530 430 200 1a “

35 340-540 440 196 1a “

36 350-550 450 196 1a “

37 360-560 460 196 1a “

38 370-570 470 196 1a “

**_______________________________________________________________________________**

**Nucleotide Genotype G.I. of**

**Alignment in middle BLAST of reference reference**

**Window No. positions of window score strain strain**

**_______________________________________________________________________________**

39 380-580 480 196 1a 2316097

40 390-590 490 196 1a “

41 400-600 500 192 1a “

42 410-610 510 188 1a “

43 420-620 520 184 1a “

44 430-630 530 184 1a “

45 440-640 540 172 1a “

46 450-650 550 172 1a “

47 460-660 560 172 1a “

48 470-670 570 172 1a “

49 480-680 580 168 1a “

50 490-690 590 168 1a “

51 500-700 600 168 1a “

52 510-710 610 168 1a “

_______________________________________________________________________________
